# NanoGBS: A Miniaturized Procedure for GBS Library Preparation

**DOI:** 10.3389/fgene.2020.00067

**Published:** 2020-02-18

**Authors:** Davoud Torkamaneh, Brian Boyle, Jérôme St-Cyr, Gaétan Légaré, Sonia Pomerleau, François Belzile

**Affiliations:** ^1^ Département de Phytologie, Université Laval, Québec City, QC, Canada; ^2^ Institut de Biologie Intégrative et des Systèmes (IBIS), Université Laval, Québec City, QC, Canada

**Keywords:** reduced-representation sequencing, genotyping, genotyping-by-sequencing, GBS library construction, NanoGBS

## Abstract

High-throughput reduced-representation sequencing (RRS)-based genotyping methods, such as genotyping-by-sequencing (GBS), have provided attractive genotyping solutions in numerous species. Here, we present NanoGBS, a miniaturized and eco-friendly method for GBS library construction. Using acoustic droplet ejection (ADE) technology, NanoGBS libraries were constructed in tenfold smaller volumes compared to standard methods (StdGBS) and leading to a reduced use of plastics of up to 90%. A high-quality DNA library and SNP catalogue were obtained with extensive overlap (96%) in SNP loci and 100% agreement in genotype calls compared to the StdGBS dataset with a high level of accuracy (98.5%). A highly multiplexed pool of GBS libraries (768-plex) was sequenced on a single Ion Proton PI chip and yielded enough SNPs (~4K SNPs; 1.5 SNP per cM, on average) for many high-volume applications. Combining NanoGBS library preparation and increased multiplexing can dramatically reduce (72%) genotyping cost per sample. We believe that this approach will greatly facilitate the adoption of marker applications where extremely high throughputs are required and cost is still currently limiting.

## Introduction

Genome-wide genotyping of large sets of samples, an essential component in a wide range of genetic studies, is greatly facilitated by genotyping polymorphic loci, also called genetic variants or markers, using next-generation sequencing (NGS)–based methods (Rasheed et al, 2017). NGS-based genotyping methods are capable of simultaneously genotyping markers on a genome-wide scale, even in nonmodel species with little or no available genetic information (Torkamaneh et al., 2018a). NGS-based reduced-representation sequencing (RRS) strategies [restriction site-associated DNA sequencing (RAD-seq), complexity reduction of polymorphic sequences (CRoPS), genotyping-by-sequencing (GBS), double-digest RAD-seq (ddRAD), 2bRAD, and double-digest GBS (ddGBS)], relying on high-throughput sequencing (HTS) of multiplexed samples, allows for the genotyping of thousands to millions of SNPs in parallel in large sets of individual samples (Baird et al., 2008; Elshire et al., 2011; Davey et al., 2011; Peterson et al., 2012; Wang et al., 2012; Ali et al., 2016; Wang et al., 2017). The RRS methods all follow similar DNA library preparation steps (DNA digestion, adaptor ligation, amplification, and sequencing) and are generically called GBS. GBS methods have been developed as rapid, high-throughput, flexible, cost-effective, and robust which make them an excellent tool for many applications and research questions (Poland and Rife, 2012; Narum et al., 2013; He et al., 2014; Andrews et al., 2016). In the last decade, GBS methods have been widely applied for genome-wide genotyping of large multiplexed samples of both model [e.g., human (Luca et al., 2011); Arabidopsis (Begali, 2018)] and nonmodel species [e.g., cattle (De Donato et al., 2013); pigs (Chen et al., 2013); maize (Elshire et al., 2011); cucumber (Zhu et al., 2016); fungi (Leboldus et al., 2015); insects (Dupuis et al., 2017); nematodes (Mimee et al., 2015)] where alternative genotyping tools (e.g., SNP arrays) are typically unavailable (Fonseca et al., 2016). The strengths and limitations of GBS methods have been comprehensively discussed and reviewed in plants (He et al., 2014), livestock (Gurgul et al., 2019), fisheries and aquaculture (Li and Wang, 2017), and ecological and conservation genomics (Narum et al., 2013).

The interest in using GBS methods for a wide range of studies is continuously growing (Andrews et al., 2016), however, they tend to present a missing data problem (Manching et al., 2017) due to low-coverage sequencing (Fu and Peterson, 2011) and variable proportions of shared loci (10%–98%), which may result in low call rates per sample (DaCosta and Sorenson, 2014). Following initial publications on GBS methods, these methods have continued to be extended and optimized in technical and computational aspects to minimize the problem of missing data (Ali et al., 2016). Technical improvements [e.g. automation of size selection (e.g. on Blue Pippin apparatus)] led to construct GBS libraries that contain fragments of similar size leading to greater uniformity in the capture of a subset of the genome (Peterson et al., 2012). On the other hand, advanced computational algorithms have enabled high-quality imputation of missing data (Torkamaneh and Belzile, 2015).

To leverage the wealth of genomic data in applied research fields (e.g., genomic selection), genotyping of large populations (thousands to millions of samples) is required. In spite of the fact that GBS methods are economical compared to array-based genotyping and significantly less expensive than whole-genome sequencing (WGS), they can nonetheless be pricey when large numbers of samples need to be analyzed, such as in breeding programs (Thomson, 2014; Gorjanc et al., 2015; Pértille et al., 2016; Rasheed et al., 2017). Despite the astounding reductions in the cost of DNA sequencing (1M-fold) over the past decade (Wetterstrand, 2018), the cost of preparing NGS libraries has not decreased as rapidly. A quick and efficient way to reduce the cost of library preparation is to reduce the reaction volume, but there is a limit to what can be achieved using standard liquid transfer approaches that rely on pipetting. Recent advances in noncontact liquid transfer approaches based on acoustic droplet ejection (ADE) technology offers a fast, accurate, uniform, and precise liquid transfer, on a nanoliter scale, which cannot be handled by humans (Hadimioglu et al., 2015).

The aim of this study was to establish an approach to significantly reduce the cost of genome-wide genotyping using GBS. To achieve this goal, we present NanoGBS, a miniaturized procedure for GBS library preparation on a nanoliter scale to minimize front-end cost. Furthermore, by increasing the multiplexing of samples in view of sequencing (768-plex per Ion Proton chip) a significant reduction in the cost of sequencing was also achieved. Our results demonstrate that, using NanoGBS and increased multiplexing, the cost of genome-wide analysis of soybean samples can be reduced by 72% while capturing the same SNP loci and genotyping with high accuracy.

## Materials and Methods

### Biological Materials

#### StdGBS vs. NanoGBS Experiment

To compare the standard GBS (StdGBS) (Elshire et al., 2011; Sonah et al., 2013) and NanoGBS methods, a set of 96 Canadian soybean lines was subjected to both protocols. These lines were selected based on the availability of WGS data (Torkamaneh et al., 2018b), a key dataset allowing for validation and quality control. Seeds were originally obtained from Dr. Istvan Rajcan lab (University of Guelph) and planted in individual 2-inch pots containing a single Jiffy peat pellet (Gérard Bourbeau & fils Inc. Quebec, Canada). The first trifoliate leaf from 12-day-old plants was harvested and immediately frozen in liquid nitrogen. Frozen leaf tissue was ground using a Qiagen Tissue Lyser. DNA was extracted from approximately 100 mg of ground tissue using the Qiagen Plant DNeasy Mini Kit according to the manufacturer's protocol. DNA was quantified on a NanoDrop spectrophotometer.

#### Large-Scale Multiplexing Experiment

To explore the impact of increased multiplexing, a set of 384 Canadian soybean lines derived from Torkamaneh et al. (2018b) was used. Three different combinations of restriction enzymes (*Ape*KI, *Pst*I*/Msp*I, and *Sbf*I/*Msp*I) and three multiplexing conditions (96-plex, 384-plex, and 768-plex (two replications of 384 lines) were used to prepare a total of nine libraries using NanoGBS. Overall, the number of SNPs, genotype quality and sequencing cost per sample were evaluated in nine different conditions.

### GBS Library Preparation

The GBS libraries based on StdGBS and NanoGBS methods were constructed following the standard protocols described by Elshire et al. (2011) and Abed et al. (2019) with an automated size selection step using a BluePippin apparatus (Sage Science, Beverley, MA, USA). The StdGBS and NanoGBS methods share all of the steps described below, with differences in volumes and liquid handling methods (**Table 1** and **Datasheet 1**).

- Genomic DNA, either 100 ng (10 µl of 10 ng/µl in StdGBS) or 10 ng (1 µl of 10 ng/µl in NanoGBS), of each sample was used for restriction digestion.
- Restriction-enzyme digestion mix [20 µl (StdGBS) vs. 2 µl (NanoGBS)] with the common restriction enzyme for soybean, *Ape*KI, was used.
- Sample-specific adapters [5 µl (StdGBS) vs. 500 nl (NanoGBS) at 0.1 μM] were added and the ligation step was done after digestion. The digestion/ligation step was thus carried out in total reaction volumes of 50 and 5 µl, respectively, for StdGBS and NanoGbS.
- In both protocols, individual libraries were pooled using 5 µl from each library. A size-selection step for both methods was done using a BluePippin (Sage Science, Beverley, MA, USA).
- PCR amplification (12 cycles), enrichment, and PCR clean-up were performed on each pool.
- Quality control, quantitation, and purity assessments for DNA was done with a spectrophotometer (Nanodrop 1000, Fisher Scientific) and a Bioanalyzer 2100 (Agilent Technologies, Santa Clara, CA, USA).
- Both pools were quantified with Picogreen and were diluted to 200 pM.
- Finally, 25 μl of each individual pool was loaded on an Ion CHEF and sequenced on an Ion Proton.

**Table 1:** Amount of reagents used for preparation of a genotyping-by-sequencing (GBS) library based on standard GBS (StdGBS) and NanoGBS methods.

GBS library prep. (µl)	StdGBS	NanoGBS
DNA	10.0	1.0
RE-digestion Mix*	20.0	2.0
Ligation Mix	15.0	1.5
Adapter†	5.0	0.5
**Total**	**50.0**	**5.0**
Dead volume‡ (µl)	5.0	1.0
Pooling (µl/sample)	5.0	5.0

*RE: restriction enzyme.

†Includes sample-specific barcodes.

‡Pipetting margin and/or residual volume that cannot be used.

During StdGBS library preparation steps i to iii, DNAs and reagents were transferred using manual and robotic pipetting (Eppendorf *epMotion* ^®^5075). For the corresponding steps of NanoGBS library preparation, an Echo^®^555 liquid handler (LABCYTE Inc.) was used for transferring and dispensing of liquids (**Supplementary Figure 1**).

### Sequencing

For the StdGBS vs. NanoGBS experiment, each 96-plex library was sequenced on a single Ion PI chip using an Ion Proton instrument. Similarly, in the multiplexing experiment, each library (96-plex, 384-plex, and 768-plex) was sequenced using a single Ion Proton PI chip. On average, each Ion Proton sequencing run yielded ~75 M single-end reads with a median length of 135 bp. Sequencing was performed by the Genomic Analysis Platform (http://www.ibis.ulaval.ca/en/services-2/genomic-analysis-platform/) at the Institut de Biologie Intégrative et des Systèmes (IBIS) of Université Laval, Quebec, Canada. An Ion CHEF (Thermo Fisher Scientific, Waltham, MA, USA) was also used for template preparation and chip loading.

### Data Analysis

Single-end sequence reads were processed using the Fast-GBS pipeline (Torkamaneh et al., 2017). In brief, FASTQ files were demultiplexed based on barcode sequences. Demultiplexed reads were trimmed and then mapped against the soybean reference genome [Williams82 (Gmax_275_Wm82.a2.v1)] (Schmutz et al., 2010). Nucleotide variants were identified from mapped reads. Variants were removed if (i) they had two or more alternate alleles, (ii) the overall base quality (QUAL) score was <10 (iii) the mapping quality (MQ) score was <30, and (iv) read depth of was <2. Finally, loci with >80% missing data were excluded. Sequencing reads, bases and genotype quality assessment were estimated using BCFtools (Li, 2011), VCFtools (Danecek et al., 2011), and TASSEL (Glaubitz et al., 2014). To estimate the accuracy of genotype calls, the resulting catalogue of variants for each method (StdGBS and NanoGBS) were compared with WGS data for the same lines from Torkamaneh et al. (2018b).

## Results and Discussion

### Miniaturized GBS Library Preparation

GBS libraries were constructed for a set of 96 soybean samples using StdGBS and NanoGBS methods. By miniaturizing GBS library preparation, the NanoGBS method saved 90% in reagent usage and reduced handling time by 75% (**Table 1**) compared to StdGBS. To minimize pipetting errors and ensure a reproducible reaction, minimum transfer volumes were fixed to 5 µl in StdGBS (Hilario, 2017; Abed et al., 2019). In contrast, a fast, accurate, uniform, and precise tipless liquid transfer, on a nanoliter scale, was achieved in NanoGBS using ADE technology (Echo^®^555 liquid handler) (Hadimioglu et al., 2015). Nevertheless, a dead volume may occur when a pool of DNA libraries is prepared, a fivefold reduction in dead volume was achieved using NanoGBS compared to StdGBS (1 vs. 5 µl). In StdGBS reactions are carried out in a final volume of 50 µl of which only 5 µl are used for pooling and 45 µl remain unused. In contrast, NanoGBS uses the whole reaction, thus making better use of all reagents. Finally, we estimate that using NanoGBS, the cost of GBS library preparation per sample can be reduced by 67% (4 C$ vs. 12 C$, including labor cost). We believe that this significant cost reduction will allow researchers to increase the sample size for genotyping.

### Quality Assessment of NanoGBS Libraries

**Figure 1:**
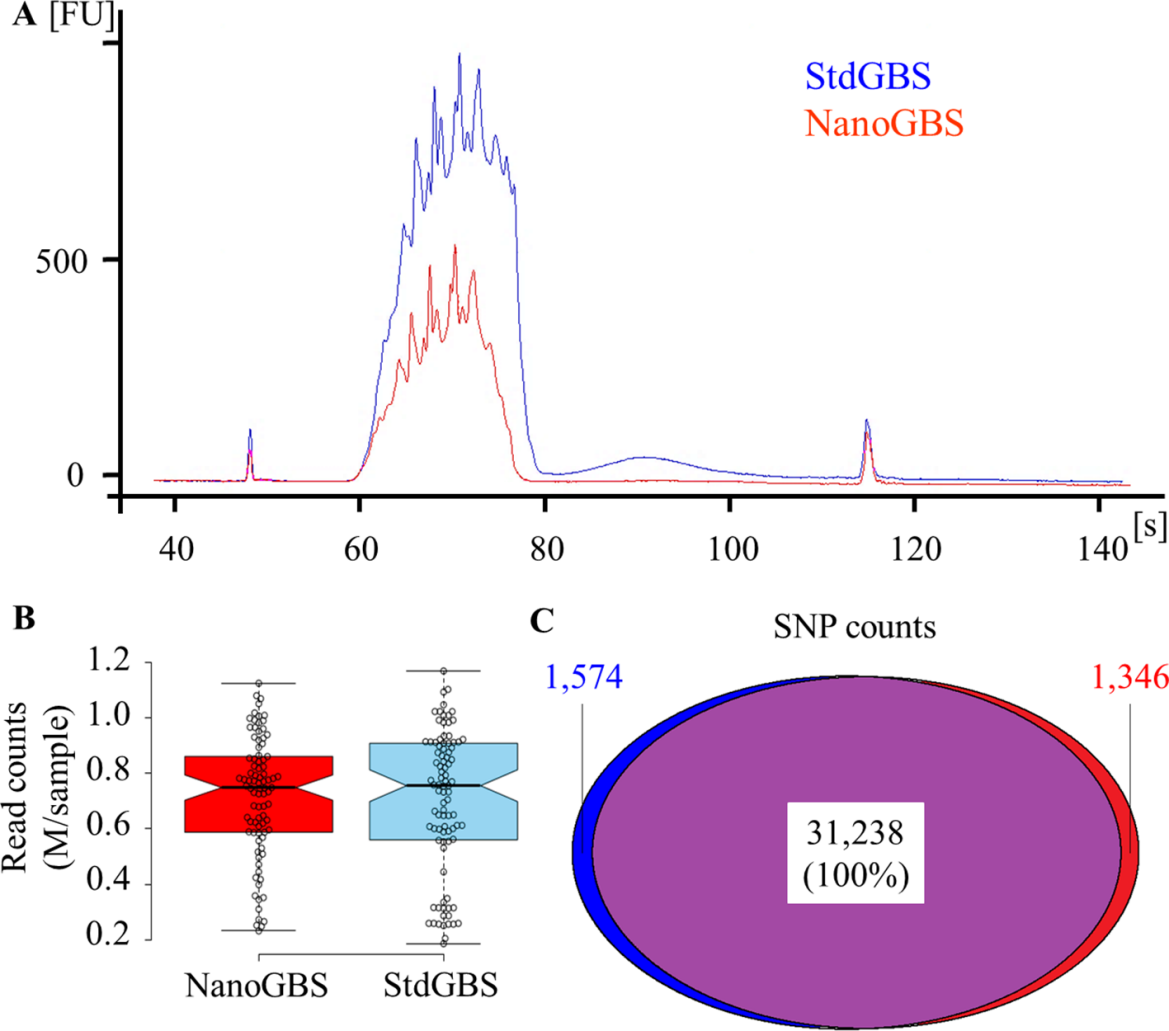
Comparison of results obtained from standard genotyping-by-sequencing (StdGBS) vs. NanoGBS pooled libraries for a set 96 soybean samples. **(A)** Quality of DNA library pools using a Bioanalyzer. **(B)** Distribution of reads per sample after demultiplexing. **(C)** Number of SNPs and overlap between SNP catalogues derived from StdGBS vs. NanoGBS libraries. The level of agreement between SNPs called with these methods presented in percentage.

A Picogreen quantification on both DNA library pools was performed, immediately after purification, and yielded an average concentration of 17 and 8 ng/μl, respectively, falling within the desired range (5–20 ng/μl) for high-quality GBS libraries (Abed et al., 2019). Even though a tenfold lower amount of DNA was used for NanoGBS library preparation, the fact that the entire reaction was used compared to 10% of the StdGBS reaction results in the same amount of DNA ultimately being used for size selection and PCR amplification. The observed difference in concentration between library preparations was most likely due to experimental variation. The quality of both DNA library pools was assessed using a Bioanalyzer trace. As can be seen in **Figure 1A**, a “Bart-Simpson hairdo” (relatively sharp edges with spikes on top) was observed for both library pools. This profile characterized by relatively sharp edges with spikes on top is expected of high-quality GBS libraries (Abed et al., 2019). No primer dimers (sharp peak at 125 bp), nor PCR overcycling effect (wide bump at > 2,000 bp) were observed in these libraries. Therefore, the miniaturization of GBS library preparation had no impact on the final quality of the libraries.

Each library pool was sequenced on an Ion Proton PI chip and generated approximately 75 million single-end reads (50–160 bp in length). Sequencing reads of pooled libraries were demultiplexed and processed with the Fast-GBS pipeline for variant calling. The uniformity of the number of reads per sample after demultiplexing is another indication of the quality of GBS libraries (Ewels et al., 2016). As can be seen in **Figure 1B**, both methods showed a similar number of reads per sample (average 0.7 M reads/sample). Interestingly, NanoGBS-derived libraries showed a greater uniformity across different samples compared to StdGBS-derived libraries (coefficient of variation of 0.32 vs. 0.37, respectively). Presumably, this greater uniformity in NanoGBS libraries can be attributed to more uniform and precise liquid handling during library preparation. Uniform sequencing of similar-sized DNA fragments in GBS libraries decreases the overall proportion of missing data (Ali et al., 2016).

Finally, the catalogues of SNPs obtained from sequencing the StdGBS and NanoGBS libraries were compared. Within the panel of 96 soybean samples, practically identical numbers of SNPs (32,812 and 32,584) were obtained with StdGBS and NanoGBS, respectively (**Figure 1C**). Not only did these SNP catalogs overlap extensively (96%), but they also presented 100% agreement in genotype calls at all loci where a call was made for the same sample with both methods. We also found 98.5% concordance between GBS and WGS genotype calls for both protocols. Furthermore, a lower proportion of missing data (38%) was achieved for NanoGBS compared to StdGBS (41%), presumably due to greater uniformity in the library preparation and sequencing. A similar proportion of heterozygous genotypes (2.3%, on average) was obtained for both methods. The fact that the resulting catalogues of SNPs overlap extensively and perfectly agree in terms of called genotypes provides strong evidence of the quality and efficacy of the NanoGBS method.

### Scalability for Large-Scale Multiplexing

**Figure 2:**
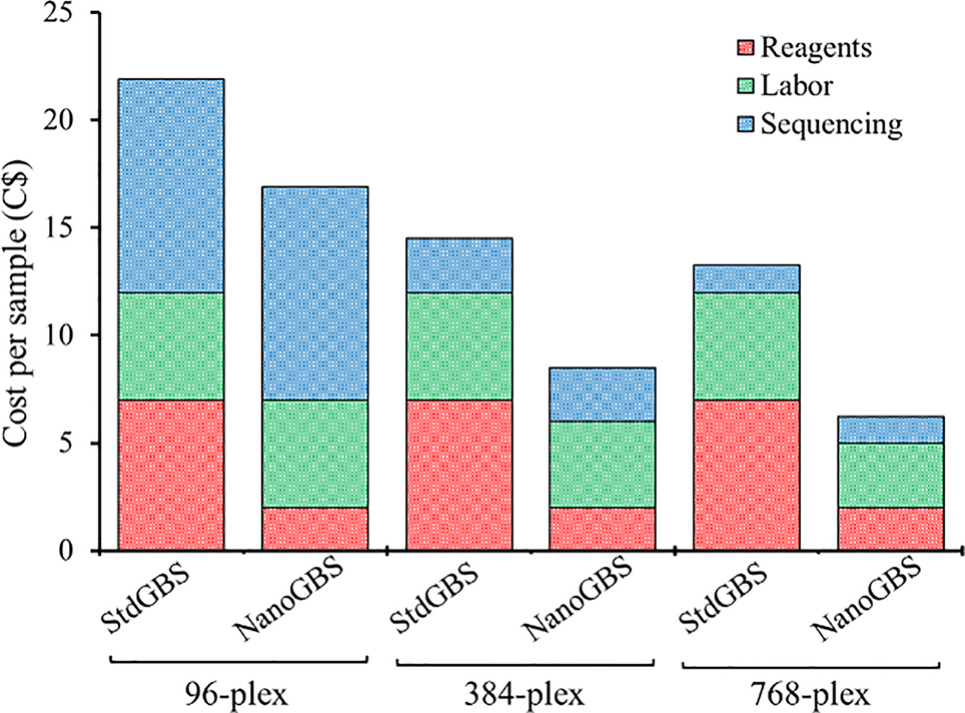
Detailed estimation of cost of genotyping-by-sequencing (GBS) genotyping per sample using standard GBS (StdGBS) vs. NanoGBS methods in three different multiplexing conditions.

Theoretically, an increased multiplexing level in GBS can be achieved through decreasing sequencing coverage or decreasing the subset of the genome that is captured during complexity reduction (e.g., by using enzymes that cut less frequently) (Torkamaneh et al., 2019). Therefore, we explored and evaluated the application of these options to increase the degree of multiplexing in GBS. Both approaches were explored by (1) increasing the multiplexing level (96-plex, 384-plex, and 768-plex) and (2) using different combinations of restriction enzymes (*Ape*KI, *Pst*I/*Msp*I, and *Sbf*I*/Msp*I). **Table 2** summarizes the results on the nine different conditions (3 multiplexing levels x 3 enzyme combinations) evaluated on the basis of estimated sequencing cost per sample and five parameters related to the genotyping quality [average read counts (million/sample), number of variants, overall proportion of missing data (%), overall proportion of heterozygous genotypes (%), and average depth of coverage (x)]. We drew three main conclusions from this. Firstly, performing GBS analysis using a frequently cutting enzyme (*Ape*KI; 4.5-base cutter) and increasing the degree of multiplexing (96- to 768-plex) dramatically reduced sequencing cost per sample (87%; 9.90C$ to 1.25C$). This occurred at the expense of the number of SNPs called (32k vs. 6k, respectively) and resulted in a higher proportion of missing data (41% vs. 67%, respectively). The larger number of samples analyzed on a single sequencing chip decreased the sequencing depth per sample and hence increased the proportion of missing data. Despite the advances in missing data imputation methods, the success of inferring missing data remains highly variable in different conditions and species (Huang and Knowles, 2016). Secondly, performing GBS analysis using a combination of an extremely infrequently cutting enzyme (*Sbf*I; 8-base cutter) and a frequently cutting enzyme (*Msp*I; 4-base cutter) resulted in a very low proportion of missing data across all multiplexing levels (5%–8%) but at the cost of a very small catalogue of SNPs (369). Finally, performing GBS using a combination of infrequently and frequently cutting enzymes (*Pst*I*/Msp*I; 6- and 4-base cutter, respectively) resulted in an appropriate number of SNPs (~4K) that is adequate for many applications (analysis of genetic diversity, genetic mapping, genomic selection), even at high multiplexing (768-plex), all the while resulting in a low proportion of missing data (24%). Typically, in GBS-derived datasets, relatively large amounts of missing data (up to 50%) can be successfully imputed (Torkamaneh and Belzile, 2015). Reducing genotyping cost per sample *via* increased sample multiplexing can make the use of molecular markers more cost-effective in various types of genetic studies, from conservation biology to breeding (Davey et al., 2011). In addition, recent studies have documented that relatively low-density genotyping can be as efficient as high-density genotyping in many cases (Raoul et al., 2016). For example, Abed et al. (2018) showed that no significant decrease in the accuracy of genomic selection was seen when using as few as ~1K SNPs compared to using 35K SNPs, even in a species such as barley that has a large genome (5.3 Gb). Gorjanc et al. (2015) explored and evaluated the impact of increased multiplexing level by decreasing sequencing coverage (from 20 to 0.05x) in livestock genomic selection populations of 500k to 50k individuals. The authors found that the accuracy of prediction was maximized when a large number of individuals were genotyped using low-coverage GBS data. Using a multiplex of 768 soybean samples, a panel of 3,756 SNPs was obtained, representing 1.5 SNP per cM, on average. This number of markers is likely sufficient for many genetic studies that would be carried out on very large numbers of individuals (Turakulov and Easteal, 2003; Habier et al., 2009). Finally, putting it all together, using NanoGBS and increasing the multiplexing level, we reduced the cost of genome-wide genotyping (~4K SNPs) in soybean by 72% overall, as a consequence of a 71% decrease in reagents, a 40% decrease in labor, and a 87% decrease in sequencing (**Figure 2**). We believe that this approach will facilitate the deployment of molecular markers in routine screening of large breeding populations and will also constitute a useful strategy for a broad array of users in different research communities.

**Table 2:** Results of genotyping-by-sequencing using different multiplexing conditions and library preparation protocols.

Multiplex*	Measured parameters	Restriction enzyme		
		ApeKI	PstI/MspI	SbfI/MspI
**96-plex**	Average read counts (million/sample)	0.9	0.9	0.9
	Number of variants	32,812	7,568	369
	Overall proportion of missing data (%)	41	12	5
	Overall proportion of heterozygous genotypes (%)	2.3	1.5	1.2
	Average depth of coverage (x)	12	33	95
	Estimated sequencing cost per sample ($)	9.90	9.90	9.90
**384-plex**	Average read counts (million/sample)	0.3	0.3	0.3
	Number of variants	18,312	3,970	369
	Overall proportion of missing data (%)	58	21	7
	Overall proportion of heterozygous genotypes (%)	1.8	1.4	1.2
	Average depth of coverage (x)	5.1	19	68
	Estimated sequencing cost per sample ($)	2.50	2.50	2.50
**768-plex**	Average read counts (million/sample)	0.1	0.1	0.1
	Number of variants	6,217	3,756	369
	Overall proportion of missing data (%)	67	24	8
	Overall proportion of heterozygous genotypes (%)	1.9	1.4	1.2
	Average depth of coverage (x)	3.7	12	44
	Estimated sequencing cost per sample ($)	1.25	1.25	1.25

*Number of samples per one Ion Proton sequencing chip.

### An Eco-Friendly GBS Library Preparation

Another novel aspect of this work is the tipless transfer approach applied in the NanoGBS method. This not only results in a dramatic reduction in cost but also significantly reduces the use of plastics (**Table 3**). In the production of a single 96-plex GBS library, we estimated that NanoGBS resulted in an eightfold reduction in the overall use of plastics (tips, plates, and tip boxes) and a fourfold reduction in nonrecyclable plastics (tips and plates) compared to StdGBS. These reductions in the amount of plastic used become all the more important when considered on a larger scale, such as the context of a genotyping service. For example, on a scale of 10,000 96-plex libraries (960,000 samples), we estimated that total reduction in plastic waste exceeds a ton. Urbina et al. (2015) estimated that world's biosciences labs could have generated as much as 5.5 MMT/Yr of plastic waste. As described, new methods like NanoGBS can contribute to dramatically reducing the plastic waste in biosciences labs. The significant reduction in the generation of plastic waste in NanoGBS not only renders this method cost-effective but also highly eco-friendly.

**Table 3:** Estimated amount of disposable plastic usage in one 96-plex genotyping-by-sequencing (GBS) experiment using standard GBS (StdGBS) and NanoGBS methods.

Library	Type of Plastic	Amount of used plastic (gr)		Reduction (%)
		StdGBS	NanoGBS	
**1**	Recyclable	63	0	100
**96-plex**	Nonrecyclable	59	14	76
	**Total**	**122**	**14**	**89**

## Strengths and Limitations

GBS methods have been widely used for simultaneous genome-wide discovery and genotyping of thousands to millions of SNPs across a wide range of species from microorganisms (Leboldus et al., 2015) to plants (He et al., 2014), insects (Dupuis et al., 2017), and animals (Gurgul et al., 2019). In large-scale applications (e.g., genomic selection and population genetics studies), plant and animal breeders, ecological and conservation geneticists need affordable and efficient genotyping tools to provide the in-depth knowledge needed to guide key decisions. Current genotyping tools (GBS, SNP arrays, and WGS) are too costly (~$25, $80, and $500/sample, respectively) for high-volume applications. NanoGBS along with an increased multiplexing level represents an interesting approach to reduce the cost of genome-wide genotyping. Here, we benchmarked NanoGBS using soybean samples, but this method could readily be used in wide range of species.

We also recognize at least two limitations to NanoGBS. First, NanoGBS relies on a highly accurate and reproducible, but fairly costly, liquid transfer technology (e.g., Echo^®^555 liquid handler). Although this technology is widely present in market, and some genomic analyses facilities have access to this technology, it could be cost-prohibitive for some GBS users. Secondly, application of a combination of different sets of restriction enzymes is not been well established for many species. Increasing multiplexing level using different set of enzymes will require additional wet-lab experiments and setups. However, to find the best enzyme combination, we recommend using bioinformatics tools [e.g., DepthFinder (Torkamaneh et al., 2019)] prior to lab experiments.

## Conclusions

GBS provides an extremely powerful and versatile tool with a wide range of applications in numerous species and fields of study. In spite of significant and continuing cost reductions in WGS, it still remains costly when used in the context of large-scale applications such as genomic selection or population diversity studies. These continued reductions in the cost of sequencing and improvements in GBS methods, such as NanoGBS, will nonetheless increase the attractiveness of GBS as a cost-effective genotyping tool in large-scale research programs. Here, we benchmarked NanoGBS using soybean samples, but this method could readily be used in wide range of species.

## Data Availability Statement

The datasets generated for this study can be found in the https://figshare.com/projects/NanoGBS_a_miniaturized_procedure_for_GBS_library_preparation/63644.

## Author Contributions

DT, BB, and FB conceptualized the concept of NanoGBS. DT, BB, JS-C, GL, and SP conducted the experiments. DT conducted data analysis. DT and FB contributed to writing the manuscript. All authors read and approved the manuscript.

## Funding

This work was supported by the SoyaGen grant (www.soyagen.ca) funded by Genome Canada [#5801 to FB]. The authors declare that this study received funding from the Génome Québec, Genome Canada, the government of Canada, the Ministère de l'Économie, Science et Innovation du Québec, Semences Prograin Inc., Syngenta Canada Inc., Sevita Genetics, Coop Fédérée, Grain Farmers of Ontario, Saskatchewan Pulse Growers, Manitoba Pulse & Soybean Growers, the Canadian Field Crop Research Alliance and Producteurs de grains du Québec. We also thank LABCYTE Inc. for providing access to an Echo^®^555 liquid handler and Dr. Charles Alex Pierson for the training. The funders were not involved in the study design, collection, analysis, interpretation of data, the writing of this article or the decision to submit it for publication.

## Conflict of Interest

The authors declare that the research was conducted in the absence of any commercial or financial relationships that could be construed as a potential conflict of interest.
