# Supplementary material for: NanoGBS: A Miniaturized Procedure for GBS Library Preparation
Source: Front Genet. 2020 Feb 18;11:67. doi: 10.3389/fgene.2020.00067 (PMC7040475; doi:10.3389/fgene.2020.00067)
Supplement: Supplementary file 1 [file DataSheet_1.docx]

**Supplementary Note.** A receipt of GBS library procedure (StdGBS and NanoGBS) for a 96-plex library.

1. **Double-stranded barcoded adapter preparation:**
   1. Suspend dried single-stranded oligonucleotides to 100 μM in elution buffer (EB).
   2. In a PCR plate, make 100 μL of 10 μM double-stranded barcoded adapters by mixing:

- 10 μL of top single-stranded oligo at 100 μM.
- 10 μL of bottom single-stranded oligo at 100 μM.
- 10 μL of 10✕ annealing buffer (AB).
- 70 μL of H_2_0.
  1. Seal the plate, mix using a mixmate, then spin down.
  2. In a thermocycler, heat to 95 °C for 1 min, then cool down to 30°C at the rate of 1°C per minute, then hold at 4 °C.
  3. Dilute 1/10 using 1✕ AB.
  4. Repeat step 4 once to bring barcoded adapters to 0.1μM.

1. **Enzyme common adapter preparation (10 μM ﬁnal):**
   1. Suspend dried single-stranded oligonucleotides to 100 μM in EB.
   2. In a PCR plate, make 100 μL of 10 μM double-stranded common adapter by mixing:

- 10 μL of top single-stranded oligo at 100 μM.
- 10 μL of bottom single-stranded oligo at 100 μM.
- 10 μL of 10✕ AB.
- 70 μL of H_2_O.
  1. Seal the plate, mix with mixmate, then spin down.
  2. In a thermocycler, heat to 95°C for 1 min and then cool at the rate of 1 °C per minute, then hold at 4 °C.

1. **Prepare working adapter plates:**
   1. Each well in the working adapter plates will have 0.02μM of a unique barcoded adapter and 1 μM of the common adapter.
   2. In a 96-well plate add:

- 20 μL Barcoded Adapters at 0.1 μM (from step 1).
- 10 μL Common Adapter at 10 μM (from step 2).
- 10 μL 10✕ AB.
- 60 μL H_2_O.
  1. Mix well and spin down.

1. **Normalize DNA and prepare sample plates:**

In DNA (sample) plates each well contains:

StdGBS

10μL of DNA at a 10 ng/μL concentration (i.e., 100 ng total).

NanoGBS

1μL of DNA at a 10 ng/μL concentration (i.e., 10 ng total).

1. **Restriction digest.**
   1. To each well of the sample plates add:

StdGBS

- - 3 μL CutSmart buffer (supplied with restriction enzymes).
  - 5 Units PstI HiFi.
  - 5 Units enzyme.
  - Complete to 30 μL with H_2_O.

NanoGBS

- - 300 nL CutSmart buffer (supplied with restriction enzymes).
  - 0.5 Units PstI HiFi.
  - 0.5 Units enzyme.
  - Complete to 3 μL with H_2_O.
  1. Mix well and spin down.
  2. Incubate in a thermocycler at 37 °C for 2 h, then hold at 8 °C.
  3. Proceed immediately with adapter ligation.

1. **Ligate adapters to cut genomic DNA:**
   1. To each well of the restriction digest plates add:

StdGBS

- - 5 μL of 10✕ T4 DNA ligase reaction buffer (supplied with T4 DNA ligase).
  - 400 Units of T4 DNA ligase.
  - 5 μL from the corresponding well of the working adapter plate.
  - Complete to 50 μL with H_2_O.

NanoGBS

- - 500 nL of 10✕ T4 DNA ligase reaction buffer (supplied with T4 DNA ligase).
  - 40 Units of T4 DNA ligase.
  - 500 nL from the corresponding well of the working adapter plate.
  - Complete to 5 μL with H_2_O.
  1. Mix well, spin down, and incubate at 22 °C for 2 h, then 65 °C for 20 min and hold at 8 °C when completed.

1. **Pooling and cleaning:**
   1. Pool 5 μL from 48 reaction wells into a 1.7 mL tube (columns 1 to 6).
   2. Repeat step 1 for the other 48 reaction wells (columns 7 to 12).
   3. Add 1.2 mL of Qiagen PB buffer to each 1.7 mL tube.
   4. Mix well using a vortex and spin down.
   5. Load 750 μL on a Qiaquick column.
   6. Spin for 15 s.
   7. Discard ﬂow-through.
   8. Repeat steps 7(7.5-7.7) until the complete volume from the two tubes has been loaded to the column.
   9. Wash column with 750μL of PE, spin 1 min, discard ﬂow-through.
   10. Rotate column and spin 1 min to remove all traces of PE.
   11. Transfer column to a new 1.7 mL tube.
   12. Add 30μL of EB to the center of the column, let stand for 1 min, then spin 1 min to elute the pooled library.
2. **Size the library using a BluePippin:**
   1. Add 10 μL of BluePippin buffer (supplied with 2% gel cassette) to the eluted library.
   2. Follow BluePippin instructions for loading on a 2% gel cassette.
   3. We set elute times from 46 to 60 min.
   4. Retrieve around 50–60 μL per library.
3. **PCR ampliﬁcation and enrichment.**
   1. Prepare of ampliﬁcation mix for each library:
   - 22.9 μL of H_2_O.
   - 10 μL of 5✕ Q5 buffer (Supplied with Q5 Polymerase).
   - 10 μL of Q5 enhancer solution (Supplied with Q5 Polymerase).
   - 1 μL of 10 mM dNTP.
   - 0.3 μL of 10 μM FWD IonExpress Primer.
   - 0.3 μL of 10 μM REV IonExpress Primer.
   - 5 μL of DNA from step 8.
   - 0.5 μL of Q5 polymerase.
   1. Mix well and spin down.
   2. Run the following PCR Program:
   - 75 °C for 5 min.
   - 5 cycles of: – 98 °C 10 s. – 55 °C 30 s. – 72 °C 30 s.
   - 7 cycles of: – 98 °C 10 s. – 65 °C 30 s. – 72 °C 30 s.
   - 72 °C 5 min.
   - Hold at 4 °C.
   1. Add 50 μL of Axygen PCR clean up kit and mix well, transfer to a 1.5 mL tube.
   2. Let stand for 5 min at room temperature.
   3. Put on magnet for 2 min.
   4. Remove the liquid without disturbing the magnetic beads.
   5. While keeping the tube on the magnet, wash the pellet twice with 1 mL of freshly prepared 80% ethanol.
   6. Remove all traces of ethanol and let dry for 10–15 min.
   7. Remove from magnet.
   8. Resuspend dried beads in 30 μL of EB, let stand for 2 min.
   9. Put on magnet and wait for 5 min for beads to pellet.
   10. Transfer your eluted library to a new tube. Be careful not to carry over beads.
4. **Quality control:**
   1. Perform a Nanodrop quantiﬁcation (expect 5 - 20 ng/μL).
   2. Bioanalyzer trace (or equivalent). There should be no primer dimers located around 100–110 nt. Background after 400 bases should be ﬂat.
   3. Quantify the library with Picogreen or equivalent. Dilute library to 200 pM.
5. **Sequencing (Ion Proton):**
   1. Follow the manufacturer’s instructions to load the Ion CHEF and Ion Proton Sequencer.
   2. Load 25 μL of a 200 pM GBS library.
   3. Run the FastqCreator plugin to generate the fastq ﬁle.
   4. Compress the fastq ﬁle using gzip.
