# Supplementary figures and images for: NanoGBS: A Miniaturized Procedure for GBS Library Preparation
Source: Front Genet. 2020 Feb 18;11:67. doi: 10.3389/fgene.2020.00067 (PMC7040475; doi:10.3389/fgene.2020.00067)

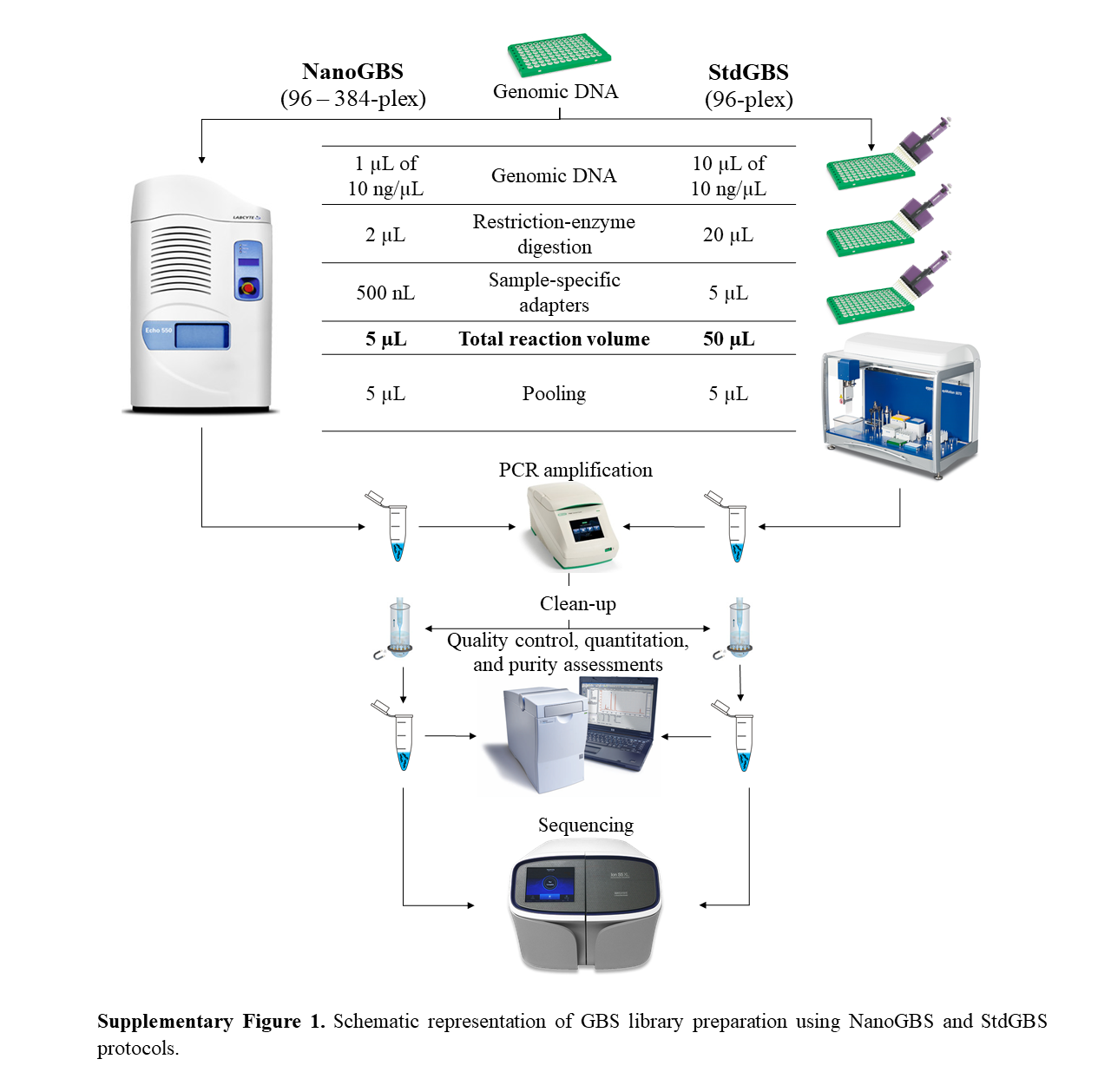

Supplement: Supplementary file 2 [file Image_1.tif]
